# Investigation of Entropy in Two-Dimensional Peristaltic Flow with Temperature Dependent Viscosity, Thermal and Electrical Conductivity

**DOI:** 10.3390/e22020200

**Published:** 2020-02-10

**Authors:** Muhammad Qasim, Zafar Ali, Umer Farooq, Dianchen Lu

**Affiliations:** 1 Department of Mathematics, Faculty of Science, Jiangsu University, Zhenjiang 212013, China; umer_sjtu@yahoo.com (U.F.); dclu@ujs.edu.cn (D.L.); 2 Department of Mathematics, COMSATS University Islamabad (CUI), 455000, Park Road, Tarlai Kalan, Islamabad 44000, Pakistan; zafarqau.bhatti@gmail.com

**Keywords:** variable fluid properties, magneto-hydrodynamics (MHD), joule dissipation, entropy generation, peristaltic flow

## Abstract

This study comprehensively explores the generalized form of two-dimensional peristaltic motions of incompressible fluid through temperature-dependent physical properties in a non-symmetric channel. Generation of entropy in the system, carrying Joule heat and Lorentz force is also examined. Viscous dissipation is not ignored, for viewing in-depth, effects of heat transmission and entropy production. The modeling of equations is tracked first in fixed and then in wave frame. The resultant set of coupled non-linear equations are solved numerically by utilizing NDSolve in Mathematica. Comparison between NDSolve and the numerical results obtained through bvp4c MATLAB is made for the validation of our numerical codes. The attained results are found to be in excellent agreement. The impact of control parameters on the velocity profiles, pressure gradient, heat transfer, streamlines and entropy production are studied and discussed graphically. It is witnessed that entropy production and heat transfer are increased significantly subject to the enhancement of Hartman number, Brinkman number and electrical conductivity parameter. Hence, choosing appropriate values of physical parameters, performance and efficiency of flow structure and system can be improved. The results reported provide a virtuous insight into bio energy systems providing a useful standard for experimental and extra progressive computational multiphysics simulations.

## 1. Introduction

Peristaltic transport is unique way in which fluid like substances are propelled inside the ducts through progressive wave trains. Basically, it is natural for the muscles and the organs of living beings to perform peristalsis in order to meet the body’s requirements. Various functions of physiology like flow of blood through the micro vessels, cilia and ovum’s movement in a fallopian tube, spermatozoa’s movement, and movement of semen in vas deferens, bile transport in a bile duct, digested food’s transport in digestive tract and urine transport are performed on the base of this mechanism. Working of lung-heart machines and blood pumps is also based on the same principle. In order to make sure that the fluid does not come into contact with machine parts it is recommended to use roller and finger pumps to propel very clean and acidic material. Therefore, peristalsis is uniquely used naturally (in living beings) and artificially in biomedical engineering and industry as well. Many studies on peristalsis are available in the literature due to its wide range of applications. Latham [1] laid the foundation to study peristaltic transport of fluids experimentally. Burns and Parkes [2] paved a way to study peristalsis and integrated wavelength and Reynolds number assumptions for a long way symmetric and asymmetric channels. Shapiro et al. [3] compared and validated the results with previous ones under the same assumptions of long wavelength and low Reynolds number. Zien and Ostrach [4] analytically studied peristalsis in two dimensions with long wave estimations for viscous incompressible fluids. Vries et al. [5] observed peristalsis exhibited by intrauterine fluid flow in symmetric and asymmetric channels because of myometrial contractions. Taylor [6] theoretically investigated the motion of microscopic organism particularly the transport of spermatozoa in asymmetric wave propagation. Carew and Pedley [7] accounted lubrication theory to study the peristaltic pumping in ureter. Some of the other prominent works were undertaken by Shukla and Gupta [8], Srivastava and Srivastava [9], Eytan and Elad [10] in which they have considered different geometries for Newtonian and non-Newtonians fluid models.

Magneto-science is currently one of the key branches of science because of its utility in medical science and biological systems. Some of its core examples are targeting drugs through magnets, magnetic cell separation devices, and rate of blood flow adjustment during surgery, transportation of bio-waste fluids, and magnet control of gastro-intestinal disorders, treatment of tumors, endoscopy procedure and technique of hyperthermia. Magnetohydrodynamics (MHD) is a branch of science that addresses the involvement of electrically conducting physiological fluids under the application of a magnetic field [11]. In medical science, bio-magnet fluids are considered as belonging to the class of physiological fluids that are highly affected by application of a magnetic field. Sud et al. [12] studied the effects of magnetic field on the blood flow rate and observed that it accelerates the speed of blood flow. Agrawal and Anwaruddin [13] studied MHD effect on the blood flow inside a branch executing peristaltic motion under long wave approximation. Mekheimer [14] applied magnetic field properties on the peristaltic flow of blood in channel. He observed that the Hartman number increases the pressure rise per wavelength. Hayat et al. [15] concluded that the Hartman number decreases the flow rate of fluid when they considered non-Newtonian fluid in channel under the stimulus of magnetic field. Abd Elmaboud [16] investigated that the magnetic number increases the pressure gradient wherein he observed peristaltic flow in an annulus with magnetic field effect. Noreen [17] studied MHD peristaltic flow with Joule heating and convective boundary conditions for couple-stress fluid. Some other prominent works [18,19,20,21] in the learning of MHD peristaltic conveyance are available in literature wherein various types of geometries and fluid models are considered.

In recent years, paramount attention has been given to the peristaltic flow of Newtonian and non-Newtonian fluids with variable physical properties. This is because, if there is a huge difference in temperature inside the medium then it is unavoidable to disregard variable viscosity, variable thermal conductivity and variable electrical conductivity. Animasaun and Oyem [22] recorded that viscosity of the fluid decreases 240% when its temperature is raised from 10 °C to 50 °C. Thus, viscosity decreases with temperature. The effect of temperature on thermal and electrical conductivity varies for different substances because of their chemical behavior and physical structure. In the case of fluids, thermal and electrical conductivity tends to increase on the upsurge of temperature because of free ions’ movement. Therefore, researchers are convinced of the importance of studying peristalsis and heat transfer with variable physical properties. Srivastava et al. [23], El Misery et al. [24], El Hakeem et al. [25], Ali et al. [26] and a few others [27,28,29,30,31] have considered variable viscosity in the peristaltic transport of fluids. In these cases, a justified relation for variable viscosity is used according to the situation of it being either space-dependent or varying with temperature. Also, it is surveyed that some authors use an exponential form of the viscosity relation and others take it to be a linear function. However, variable thermal conductivity is scarcely accounted for in the previous works. Qamar et al. [32] investigated Jeffrey fluid with peristalsis by taking both viscosity and thermal conductivity to be functions of temperature. Qamar et al. [33] again considered variable thermal conductivity with MHD Jeffrey fluid with heat transfer in peristalsis. Mekheimer and Elmaboud [34] investigated peristalsis with heat transfer under the influence of variable viscosity and thermal conductivity in a vertical asymmetric channel. Hayat et al. [35] obtained numerical results for the effects of variable viscosity and variable thermal conductivity along with Ohmic heating in an inclined asymmetric channel. In all the above works, electrical conductivity is continuously taken as a constant property of the fluid. However, its variable effects are ignored when the flow is accompanied with heat transfer under the effects of Lorentz strength and Joule heating. Its negligence might be because of the fact that if all properties are taken variable then the reduced coupled partial differential equations are highly non-linear as they are not easy to handle with ordinary methods.

Entropy generation, in thermodynamics, measures the heat transfer irreversibility of the system. System performance badly suffers during the engineering process because of the irreversibility loss of energy. It is accepted generally that the thermal irreversibility and energy losses due to frictional forces, temperature difference, viscosity, chemical reaction and so forth, are determined by entropy generation. Therefore, minimizing entropy generation and increasing machines efficiency is greatly desired, and this has influenced the scientists and researchers. Thus, in optimization of system irreversibility, the second law of thermodynamics was deemed to be more efficient compared to the first law and entropy generation is the only way to handle it. Bejan [36] confirmed that the heat transfer and viscous shear stresses are responsible for entropy generation. Bejan [37] and Abbasi [38] also proposed that the irreversibility losses can be acquired and examined by entropy generation which eventually supplies appropriate implements for optimization analysis, especially for the modeling of fluid flow and heat transfer devices. Abu-Hijleh [39] numerically investigated natural convection and entropy generation analysis when fluid flows through a horizontal cylinder. Other researches [40,41,42] have contributed a lot in examining the entropy generation with boundary layer flows. Entropy analysis with peristalsis is also a hot research area of this age. A significant amount work has been done in this area as well. Qasim et al. [43] carried out the entropy analysis of nanofluids flowing sinusoidal in a channel. Moreover, Noreen et al. [44] studied the entropy creation in electro-osmotically peristaltically curving viscous fluid. Abbas et al. [45] studied the second law analysis for the peristaltic motion of nanofluid inside a non-uniform channel with compliant walls. Maraj [46] theoretically studied the entropy generation analysis contributed by heat transfer and fluid viscosity during the peristaltic flow of nanofluid in an asymmetric channel. Munawar et al. [47] attempted to investigate the effects of variable viscosity on the peristaltic flow and entropy generation of fluid. Narla et al. [48] applied the second law to investigate entropy generation for the peristaltic flow of incompressible fluid in a curved channel. Hayat et al. [49] carried out entropy analysis of nanofluid with mixed convection and peristalsis. Ranjit and Shit [50] studied the entropy generation for the peristaltic flow of biofluid in a micro-channel.

In view of the above literature, the authors are convinced that no such work has been carried out yet in which entropy generation analysis has been observed for the peristaltic flow of variable properties fluid. This paper also focuses on all variable properties including electrical conductivity with peristalsis and entropy generation in an asymmetric channel. Lorentz force and Joule heating are thus dominating factors in this problem; these effects contribute variable electrical conductivity in momentum, energy equations and entropy generation equations. Owing to complexity and non-linearity in resultant system equations, numerical methods are used to solve this problem. Graphical results are further plotted to understand the behavior of varying flow properties on velocity profile, pressure gradient and streamlines based on the variation of appearing parameters.

## 2. Mathematical Formulation

In the presence of mixed convection, consider the two-dimensional flow of an electrically conducting fluid flowing through an asymmetric channel of width $d_{1}+d_{2}$. Fluid is considered to move along the centre line of the channel in length direction, i.e., along $\bar{X}-axis$. It is further assumed that, due to low magnetic Reynolds number, an induced magnetic field is not considered so a uniform magnet field $B_{0}$ is applied parallel to the $\bar{Y}-axis$. Physical and thermal properties like viscosity, thermal conductivity and electrical conductivity are taken to be temperature dependent. The temperature of the lower and upper bounds of the conduit are presumed to be ${\bar{T}}_{1}$ and $\bar{T_{0}}$, where ${\bar{T}}_{1}>\bar{T_{0}}$. The peristaltic wave moves on walls of the channel with speed s along its length. Therefore, shape of the walls is given as:

(1) $${\bar{W}}_{1}\;=\;d_{1}+a_{1}cos\left[\frac{2\pi }{\lambda }\left(\bar{X}-c\bar{t}\right)\right]$$

(2) $${\bar{W}}_{2}\;=\;-d_{2}-a_{2}cos\left[\frac{2\pi }{\lambda }\left(\bar{X}-c\bar{t}\right)+\phi \right]$$

To ensure that the waves are not intersecting with each other $a_{1},\;a_{2},\;d_{1},\;d_{2}$ must meet the condition $\left[a_{1}^{2}+a_{2}^{2}+2a_{1}a_{2}cos\left(\phi \right)\right]\leq {\left(d_{1}+\;d_{2}\right)}^{2}$, with (ϕ+π) the phase difference, limiting 0≤ϕ≤π. Also, the symmetry of the channel depends on ϕ, where ϕ = 0 corresponds to a symmetric channel with waves out of phase and ϕ = π is taken for waves in phase. Equations of continuity, motion and energy in a fixed frame are:

(3) $$\frac{\partial \bar{U}}{\partial \bar{X}}+\frac{\partial \bar{V}}{\partial \bar{Y}}\;=\;0$$

(4) $$\rho \left[\frac{\partial \bar{U}}{\partial \bar{t}}+\bar{U}\frac{\partial \bar{U}}{\partial \bar{X}}+\bar{V}\frac{\partial \bar{U}}{\partial \bar{Y}}\right]\;=\;-\frac{\partial \bar{P}}{\partial \bar{X}}+2\frac{\partial }{\partial \bar{X}}\left[\bar{\mu }\left(\bar{T}\right)\frac{\partial \bar{U}}{\partial \bar{X}}\right]+\frac{\partial }{\partial \bar{Y}}\left[\bar{\mu }\left(\bar{T}\right)\left(\frac{\partial \bar{V}}{\partial \bar{X}}+\frac{\partial \bar{U}}{\partial \bar{Y}}\right)\right]-\bar{\sigma }\left(\bar{T}\right)B_{0}^{2}\bar{U}$$

(5) $$\rho \left[\frac{\partial \bar{V}}{\partial \bar{t}}+\bar{U}\frac{\partial \bar{V}}{\partial \bar{X}}+\bar{V}\frac{\partial \bar{V}}{\partial \bar{Y}}\right]\;=\;-\frac{\partial \bar{P}}{\partial \bar{Y}}+2\frac{\partial }{\partial \bar{Y}}\left[\bar{\mu }\left(\bar{T}\right)\frac{\partial \bar{V}}{\partial \bar{Y}}\right]+\frac{\partial }{\partial \bar{X}}\left[\bar{\mu }\left(\bar{T}\right)\left(\frac{\partial \bar{V}}{\partial \bar{X}}+\frac{\partial \bar{U}}{\partial \bar{Y}}\right)\right]$$

(6) $$\rho c_{p}\left[\frac{\partial \bar{T}}{\partial \bar{t}}+\bar{U}\frac{\partial \bar{T}}{\partial \bar{X}}+\bar{V}\frac{\partial \bar{T}}{\partial \bar{Y}}\right]\;=\;\frac{\partial }{\partial \bar{X}}\left(\bar{k}\left(\bar{T}\right)\frac{\partial \bar{T}}{\partial \bar{X}}\right)+\frac{\partial }{\partial \bar{Y}}\left(\bar{k}\left(\bar{T}\right)\frac{\partial \bar{T}}{\partial \bar{Y}}\right)+\bar{\mu }\left(\bar{T}\right)\Phi +\bar{\sigma }\left(\bar{T}\right)B_{0}^{2}{\bar{U}}^{2}$$

here $\Phi \;=\;2\left\{{\left(\frac{\partial \bar{U}}{\partial \bar{X}}\right)}^{2}+{\left(\frac{\partial \bar{V}}{\partial \bar{Y}}\right)}^{2}\right\}+{\left(\frac{\partial \bar{V}}{\partial \bar{X}}+\frac{\partial \bar{U}}{\partial \bar{Y}}\right)}^{2}$ represents the viscous dissipation in (6) and $\bar{\mu }\left(\bar{T}\right)\;=\;\mu_{0}\left[1-\alpha_{1}\left(\bar{T}-{\bar{T}}_{0}\right)\right],\;\bar{k}\left(\bar{T}\right)\;=\;k_{0}\left[1+\beta_{1}\left(\bar{T}-{\bar{T}}_{0}\right)\right],\;\bar{\sigma }\left(\bar{T}\right)\;=\;\sigma_{0}\left[1+\gamma_{1}\left(\bar{T}-{\bar{T}}_{0}\right)\right]$, respectively are the relations for temperature dependent viscosity [26,27,28,29,30,31], thermal conductivity [32,33,34,35] and electrical conductivity [51].

Following the trend of transforming equations from fixed frame $\left(\bar{X},\bar{Y}\right)$ to the wave frame $\left(\bar{x},\bar{y}\right)$ we introduce:

(7) $$\bar{U}\left(\bar{X},\bar{Y},\;\bar{t}\right)\;=\;\bar{u}\left(\bar{x},\bar{y}\right),\;\bar{\;V}\left(\bar{X},\bar{Y},\;\bar{t}\right)\;=\bar{\;v}\left(\bar{x},\bar{y}\right),\;\bar{P}\left(\bar{Y},\bar{t}\right)\;=\;\bar{p}\left(\bar{y}\right),\;\bar{\;X}=\bar{x}+c\bar{t},\;\bar{Y}\;=\bar{\;y}$$

Utilizing (7) in (3)–(6) in the above Equations

(8) $$\frac{\partial \bar{u}}{\partial \bar{x}}+\frac{\partial \bar{v}}{\partial \bar{y}}\;=\;0,$$

(9) $$\rho \left[\left(\bar{u}+c\right)\frac{\partial \bar{u}}{\partial \bar{x}}+\bar{v}\frac{\partial \bar{u}}{\partial \bar{y}}\right]\;=\;-\frac{\partial \bar{p}}{\partial \bar{x}}+2\frac{\partial }{\partial \bar{x}}\left[\bar{\mu }\left(\bar{T}\right)\frac{\partial \bar{u}}{\partial \bar{x}}\right]+\frac{\partial }{\partial \bar{y}}\left[\bar{\mu }\left(\bar{T}\right)\left(\frac{\partial \bar{v}}{\partial \bar{x}}+\frac{\partial \bar{u}}{\partial \bar{y}}\right)\right]-\bar{\sigma }\left(\bar{T}\right)B_{0}^{2}\left(\bar{u}+c\right),$$

(10) $$\rho \left[\left(\bar{u}+c\right)\frac{\partial \bar{v}}{\partial \bar{x}}+\bar{v}\frac{\partial \bar{v}}{\partial \bar{y}}\right]\;=\;-\frac{\partial \bar{p}}{\partial \bar{Y}}+2\frac{\partial }{\partial \bar{y}}\left[\bar{\mu }\left(\bar{T}\right)\frac{\partial \bar{v}}{\partial \bar{y}}\right]+\frac{\partial }{\partial \bar{x}}\left[\bar{\mu }\left(\bar{T}\right)\left(\frac{\partial \bar{v}}{\partial \bar{x}}+\frac{\partial \bar{u}}{\partial \bar{y}}\right)\right],$$

(11) $$\begin{array}{c} \rho c_{p}\left[\left(\bar{u}+c\right)\frac{\partial \bar{T}}{\partial \bar{x}}+\bar{v}\frac{\partial \bar{T}}{\partial \bar{y}}\right]\\ \;\;\;\;\;\;=\;\frac{\partial }{\partial \bar{x}}\left(\bar{k}\left(\bar{T}\right)\frac{\partial \bar{T}}{\partial \bar{x}}\right)+\frac{\partial }{\partial \bar{y}}\left(\bar{k}\left(\bar{T}\right)\frac{\partial \bar{T}}{\partial \bar{y}}\right)+\bar{\mu }\left(\bar{T}\right)\left[2\left\{{\left(\frac{\partial \bar{u}}{\partial \bar{x}}\right)}^{2}+{\left(\frac{\partial \bar{v}}{\partial \bar{y}}\right)}^{2}\right\}+{\left(\frac{\partial \bar{v}}{\partial \bar{x}}+\frac{\partial \bar{u}}{\partial \bar{y}}\right)}^{2}\right]\\ \;\;\;\;\;\;+\bar{\sigma }\left(\bar{T}\right)B_{0}^{2}{\left(\bar{u}+c\right)}^{2}. \end{array}$$

Introducing the non-dimensional parameters:

(12) $$\begin{array}{c} \bar{x}\;=\;x\lambda ,\;\bar{y}\;=\;yd_{1},\bar{u}\;=\;uc\;,\;\bar{v}=vc\delta ,\;\bar{p}\;=\;\left(c\lambda \mu /d_{1}^{2}\right)p,\;\theta \;=\;\left(\bar{T}-\bar{T_{0}}\right)/\left({\bar{T}}_{1}-\bar{T_{0}}\right),\\ \bar{\psi \;}=\;\psi /\left(cd_{1}\right),\;u\;=\;\partial \psi /\partial y,\;v\;=\;-\delta \partial \psi /\partial x,\;\delta \;=\;d_{1}/\lambda ,\;w_{1}\;=\;{\bar{W}}_{1}/d_{1},\;w_{2}\;=\;{\bar{W}}_{2}/d_{1},\\ \;d\;=\;d_{2}/d_{1},a\;=\;a_{1}/d_{1},\;\;b\;=\;\;a_{2}/d_{1},\;\;Re\;=\;\rho cd_{1}/\mu_{0},\;Ha=\;B_{0}d_{1}\sqrt{\sigma_{0}/\mu_{0}},\;Br\;=\\ \;\mu_{0}c^{2}/\left[k\left({\bar{T}}_{1}-\bar{T_{0}}\right)\right],\;\alpha \;=\;\alpha_{1}\left({\bar{T}}_{1}-\bar{T_{0}}\right),\;\;\beta \;=\;\beta_{1}\left({\bar{T}}_{1}-\bar{T_{0}}\right),\;\;\gamma \;=\;\gamma_{1}\left({\bar{T}}_{1}-\bar{T_{0}}\right) \end{array}$$

Thus, using (12) and after employing long wavelength and low Reynolds number approximation [2], (8)–(11) reduces to:

(13) $$\frac{\partial p}{\partial x}\;=\;\frac{\partial }{\partial y}\left[\mu \left(\theta \right)\frac{\partial^{2}\psi }{\partial y^{2}}\right]-{\left(Ha\right)}^{2}\sigma \left(\theta \right)\left(\frac{\partial \psi }{\partial y}+1\right)$$

(14) $$\frac{\partial p}{\partial y}\;\;=\;\;0$$

(15) $$\frac{\partial }{\partial y}\left[k\left(\theta \right)\frac{\partial \theta }{\partial y}\right]+\mu \left(\theta \right)Br{\left(\frac{\partial^{2}\psi }{\partial y^{2}}\right)}^{2}+\sigma \left(\theta \right)Br{\left(Ha\right)}^{2}{\left(\frac{\partial \psi }{\partial y}+1\right)}^{2}\;=\;0,$$

On eliminating pressure gradient, momentum and heat equations become

(16) $$\frac{\partial^{2}}{\partial y^{2}}\left[\mu \left(\theta \right)\frac{\partial^{2}\psi }{\partial y^{2}}\right]-\frac{\partial }{\partial y}\left\{{\left(Ha\right)}^{2}\sigma \left(\theta \right)\left(\frac{\partial \psi }{\partial y}+1\right)\right\}\;=\;0,$$

(17) $$\frac{\partial }{\partial y}\left[k\left(\theta \right)\frac{\partial \theta }{\partial y}\right]+\mu \left(\theta \right)Br{\left(\frac{\partial^{2}\psi }{\partial y^{2}}\right)}^{2}+\sigma \left(\theta \right)Br{\left(Ha\right)}^{2}{\left(\frac{\partial \psi }{\partial y}+1\right)}^{2}\;=\;0.$$

In Equations (16) and (17) relations for temperature dependent viscosity [26,27,28,29,30,31], thermal conductivity [32,33,34,35] and electrical conductivity [51] are defined as

(18) μ(θ) = (1−αθ),  k(θ) = (1+βθ),  σ(θ) = (1+γθ).

Deriving the relations for boundary condition, let in fixed frame the volumetric flow rate is defined as:

(19) $$Q\;=\;\int_{{\bar{w}}_{2}\left(\bar{X,}\bar{t}\right)}^{{\bar{w}}_{1}\left(\bar{X,}\bar{t}\right)}\bar{U}\left(\bar{X},\bar{Y},\bar{t}\right)d\bar{Y}$$

and in the wave frame it is defined as:

(20) $$q\;=\;\int_{{\bar{w}}_{2}\left(\bar{x}\right)}^{{\bar{w}}_{1}\left(\bar{x}\right)}\bar{u}\left(\bar{x},\bar{y}\right)d\bar{y}$$

where we know that ${\bar{h}}_{1}$ and ${\bar{h}}_{2}$ are only functions of $\bar{x}$, hence using (12), (19) and (20), we have

(21) $$Q\;=\;q+c{\bar{w}}_{1}\left(\bar{x}\right)-c{\bar{w}}_{2}\left(\bar{x}\right).$$

Defining the average time flow in a T period at fixed position $\bar{X}$ is

(22) $$\bar{Q}\;=\;\frac{1}{T}\int_{0}^{T}Qdt.$$

On making use of (21) in (22) we get

(23) $$\bar{Q}\;=\;q+cd_{1}+cd_{2}$$

Defining mean flow rates as

(24) $$\theta^{*}\;=\frac{\bar{Q}}{c\;d_{1}}\;\mathrm{And}\;F\;=\;\frac{q}{cd_{1}}.$$

Thus, in laboratory and wave frames respectively, we have

(25) $$\theta^{*}\;=\;F+1+d$$

Finally, we reached boundary conditions of the form

(26) $$\psi \;=\;\frac{F}{2},\;\frac{\partial \psi }{\partial y}\;=\;-1\;\mathrm{and}\;\theta \;=\;0\;\mathrm{at}\;y\;=\;w_{1}\left(x\right)\;=\;1+acos2\pi x$$

(27) $$\psi \;=\;-\frac{F}{2},\;\frac{\partial \psi }{\partial y}\;=\;-1\;\mathrm{and}\;\theta \;=\;1\;\mathrm{at}\;y\;=\;w_{2}\left(x\right)\;=\;-d-bcos\left(2\pi x+\phi \right)$$

Pressure rise per wavelength and friction forces across the unit wavelength at both walls are

(28) $$\Delta P_{\lambda }\;=\;\int_{0}^{1}\frac{dp}{dx}dx,$$

For an incompressible Newtonian fluid flow, the volumetric local entropy generation rate is given as:

(29) $${\dot{E}}_{G}^{\prime \prime \prime }\;=\;\frac{\bar{k}\left(\bar{T}\right)}{{\bar{T}}^{2}}\left[{\left(\frac{\partial \bar{T}}{\partial \bar{X}}\right)}^{2}+{\left(\frac{\partial \bar{T}}{\partial \bar{Y}}\right)}^{2}\right]+\frac{\bar{\mu }\left(\bar{T}\right)}{\bar{T}}\left[2\left\{{\left(\frac{\partial \bar{U}}{\partial \bar{X}}\right)}^{2}+{\left(\frac{\partial \bar{V}}{\partial \bar{Y}}\right)}^{2}\right\}+{\left(\frac{\partial \bar{V}}{\partial \bar{X}}+\frac{\partial \bar{U}}{\partial \bar{Y}}\right)}^{2}\right]+\frac{\bar{\sigma }\left(\bar{T}\right)}{\bar{T}}B_{0}^{2}{\bar{U}}^{2}$$

First, second and third terms of (30) generate entropy in dimensional form owed to heat flow, fluid resistance irreversibility and magnetic field respectively.

A distinctive (traditionally known as characteristic) entropy generation is given by

(30) $${\left({\dot{E}}_{G}^{\prime \prime \prime }\right)}_{0}\;=\;\frac{k_{0}}{d_{1}^{2}}$$

Utilizing (29), (30) and (12), the expression of entropy generation number in dimensionless form as:

(31) $$D_{E}\;=\;\frac{{\dot{E}}_{G}^{\prime \prime \prime }}{{\left({\dot{E}}_{G}^{\prime \prime \prime }\right)}_{0}}\;=\;\left[\frac{\left(1+\beta \theta \right)}{{\left(\theta +\wedge \right)}^{2}}{\left(\frac{\partial \theta }{\partial y}\right)}^{2}+B_{r}\frac{\left(1-\alpha \theta \right)}{\left(\theta +\wedge \right)}{\left(\frac{\partial^{2}\psi }{\partial y^{2}}\right)}^{2}+{\left(Ha\right)}^{2}B_{r}\frac{\left(1+\gamma \theta \right)}{\left(\theta +\wedge \right)}{\left(\frac{\partial \psi }{\partial y}+1\right)}^{2}\right]$$

where ∧ is the temperature difference parameter defined by

(32) $$\wedge \;=\;\frac{T_{0}}{△T}$$

Therefore, total entropy generation can be written as:

(33) $$D_{E\;}=\;D_{T}+D_{F}+D_{JM}$$

here in (33) $D_{T}$ represents entropy generation because of heat transfer, $D_{F}$ for entropy production by reason of fluid friction irreversibility and the entropy produced for Joule heating and magnetic field is represented by $D_{JM}$.

Bejan number [37,38] or the irreversibility ratio is measure of the relative contribution entropy generations due to heat and other factors like fluid friction and magnetic field thus can be well-defined as the relation of heat transmission irreversibility to the total irreversibility, mathematically:

(34) $$Be=\frac{D_{T}}{D_{E}}$$

(35) $$Be\;\;=\;\;\frac{\frac{\left(1+\beta \theta \right)}{{\left(\theta +\wedge \right)}^{2}}{\left(\frac{\partial \theta }{\partial y}\right)}^{2}}{\left[\frac{\left(1+\beta \theta \right)}{{\left(\theta +\wedge \right)}^{2}}{\left(\frac{\partial \theta }{\partial y}\right)}^{2}+B_{r}\frac{\left(1-\alpha \theta \right)}{\left(\theta +\wedge \right)}{\left(\frac{\partial^{2}\psi }{\partial y^{2}}\right)}^{2}+{\left(Ha\right)}^{2}B_{r}\frac{\left(1+\gamma \theta \right)}{\left(\theta +\wedge \right)}{\left(\frac{\partial \psi }{\partial y}+1\right)}^{2}\right]}$$

Be∈[0,1], therefore, correlation between irreversibility of fluid friction heat transfer depends on the values of Bejan number, thus for

$$Be\;=\;\{\begin{array}{cc} 0,\;\mathrm{All}\mathrm{other}\mathrm{irreversibilities}\mathrm{dominates}\mathrm{heat}\mathrm{transfer}\mathrm{irreversibility} & \\ \frac{1}{2},\;\;\;\mathrm{All}\mathrm{irreversibilities}\mathrm{equals}\mathrm{heat}\mathrm{transfer}\mathrm{irreversibility} & \\ 1,\;\;\;\;\;\;\;\;\mathrm{Heat}\mathrm{flow}\mathrm{irreversibility}\mathrm{dominates} & \end{array}$$

## 3. Solution Methodology

The numerical solution of a nonlinear system of differential Equations (16) and (17) along with boundary conditions (26) and (27) is obtained by Mathematica through NDSolve [35,43,47,52,53,54]. The numerical solutions are also computed by using Matlab built-in boundary value solver bvp4c. Matlab code bvp4c for two-point boundary value problem is a finite difference code based on three-stage collocation at Lobatto points [55]. Table 1 shows the comparison of present results with the existing study [56]. The comparison between NDSolve and bvp4c is presented in Table 2 for the validation of our numerical codes. The achieved results are found to be in excellent agreement.

## 4. Discussion

This section is devoted for the outcomes of analysis of various parameters appeared so far in the flow and the irreversibility examination. Behavioral analysis of flow field, temperature profile, entropy generation and Bejan number subject to Hartman number $\left(Ha^{2}\right)$, variable viscosity parameter (α), variable electrical conductivity parameter (γ), variable thermal conductivity parameter (β) and Brinkman number (Br) is presented below.

**Figure 1:**
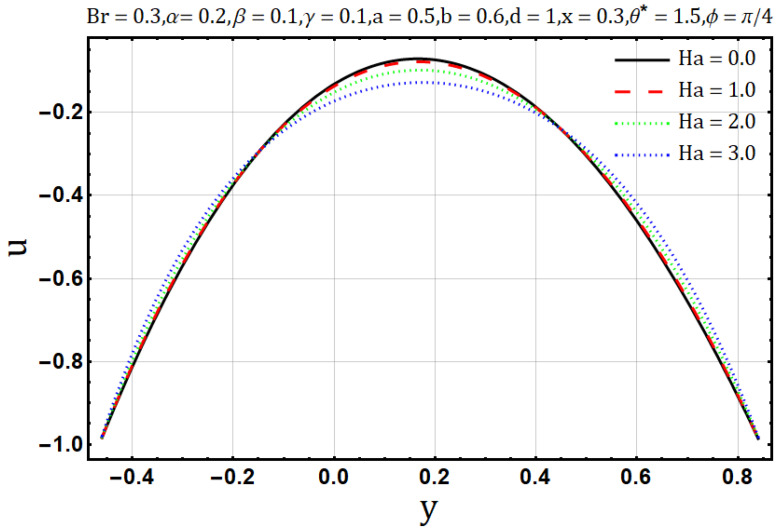
Velocity profile for Hartman number (Ha).

**Figure 2:**
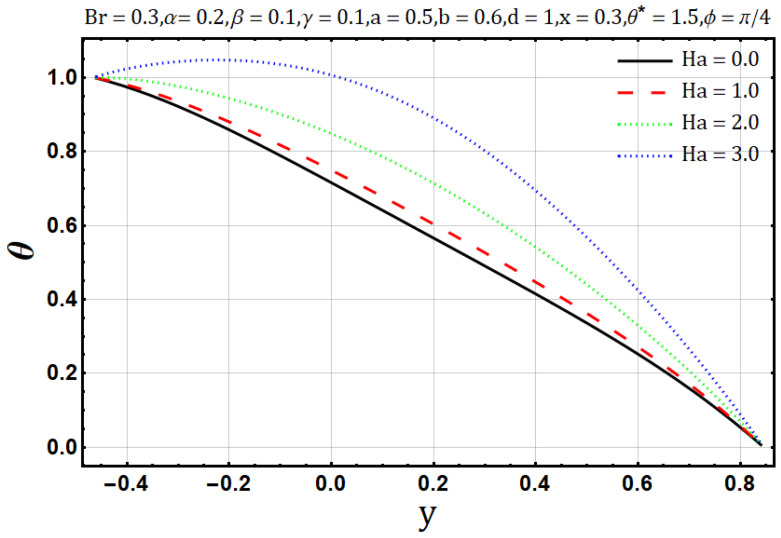
Temperature profile for Hartman number (Ha).

**Figure 3:**
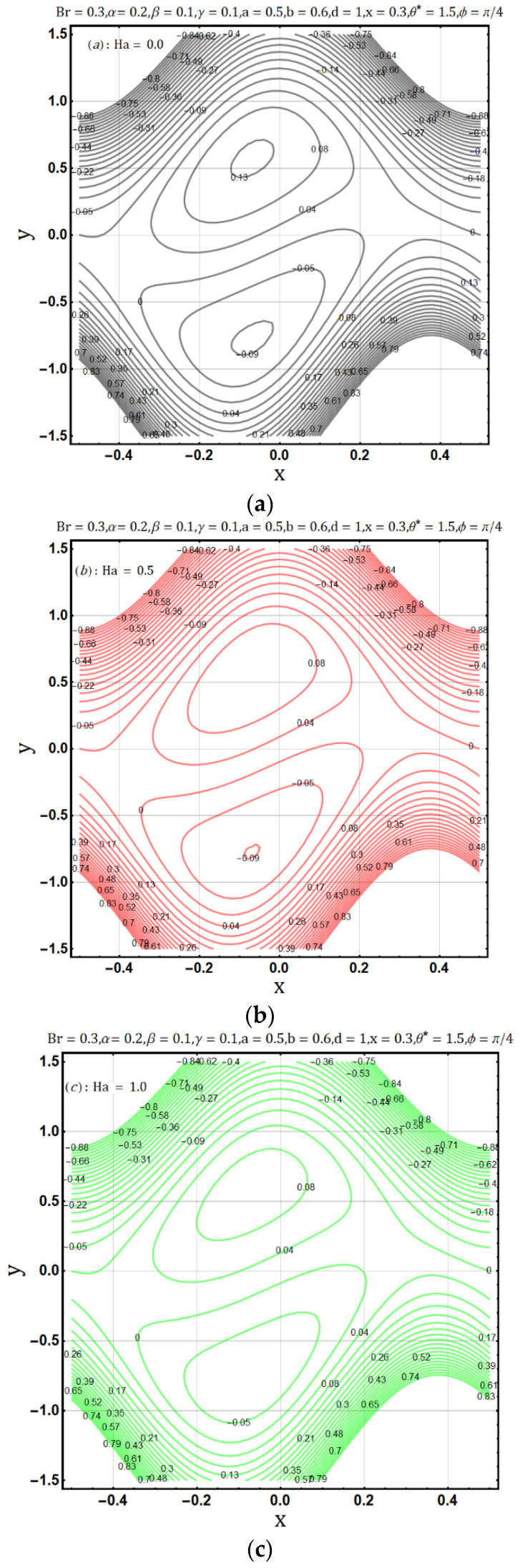
(**a**): Influence of Hartman number (Ha = 0.0) on trapping. (**b**): Influence of Hartman number (Ha = 0.5) on trapping. (**c**): Influence of Hartman number (Ha = 0.0) on trapping.

**Figure 4:**
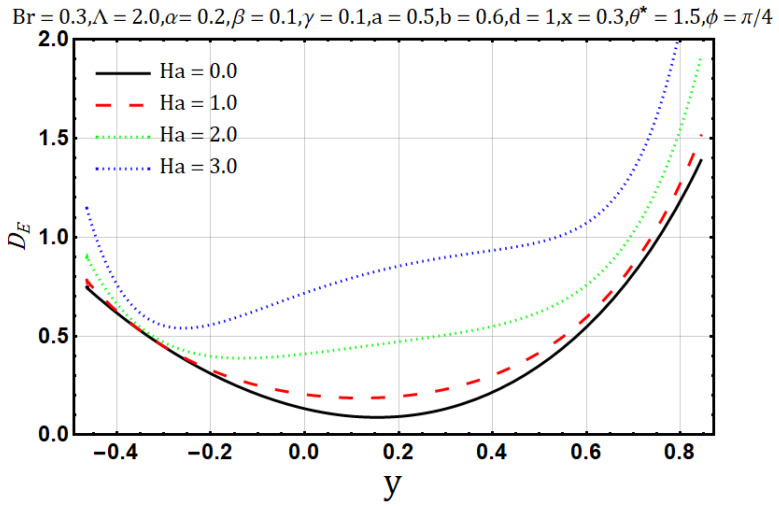
Entropy Generation Profile for Hartman Number (Ha).

**Figure 5:**
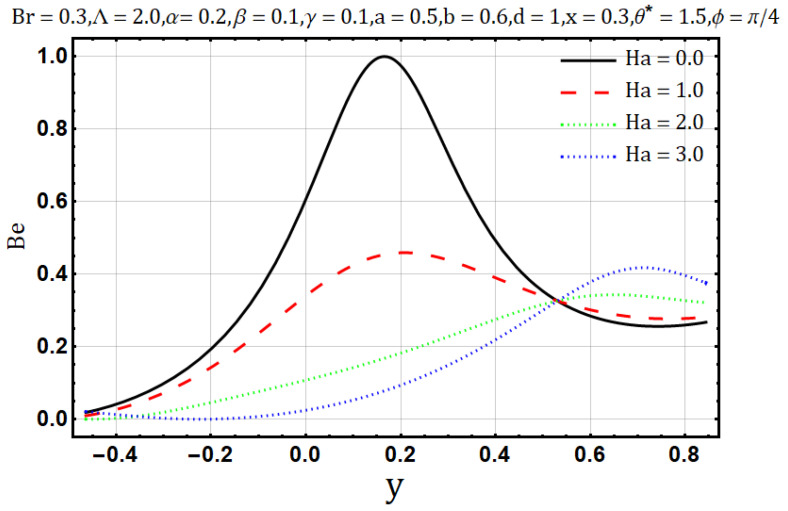
Bejan number profile for Hartman number (Ha).

Influence of Hartman number $\left(Ha^{2}\right)$:

Hartman number appears in analysis due to magnetic fields applied in the fluid flow and it gives the measurement of relative forces arising due to magnetic and viscous forces. Figure 1 shows the outcome of Hartman number $\left(Ha^{2}\right)$ on velocity profile, wherein it is observed that the velocity is dominated by the viscous and magnetic forces, hence it tends to decrease when the Hartman number $\left(Ha^{2}\right)$ is increased but near the channel walls the aforementioned forces are dominated by the velocity profile and present opposite behavior. In Figure 2, it is observed that the temperature profile is boosted due to an increase in Hartman number $\left(Ha^{2}\right)$, thus heat flow and Hartman number $\left(Ha^{2}\right)$ are found to be in good agreement. A unique hydrodynamic property associated to peristaltic transport is *trapping* which occasionally occurs while subject to large amplitude ratio. In laboratory frame, the set of streamlines represent a fluid bolus moving with and within the wave and also while streamlines bypass the trapped bolus they attain the shape similar to that of the wall’s shape. Figure 3 is to analyze the effect of Hartman number $\left(Ha^{2}\right)$ on the trapped bolus. Through (3a-3c) it is evident that the size of trapped bolus shrinks and number of boli(boluses) decreases on increasing values of Hartman number $\left(Ha^{2}\right)$ because of weak streamlines circulations. Thus, magnetic field force can be used to control the bolus formations. Figure 4 shows the effect of Hartman number $\left(Ha^{2}\right)$ on entropy generation $D_{E}$, wherein it is observed that the entropy generation $D_{E}$ increases with the increasing strength of the magnetic field. A measure of the irreversibility ratio or the Bejan number Be with respect to Hartman number $\left(Ha^{2}\right)$ is seen in Figure 5, as the Hartman number $\left(Ha^{2}\right)$ increases in magnitude, the irreversibility ratio or the Bejan number Be is seen decreasing in the center of channel but increasing near the channel walls.

**Figure 6:**
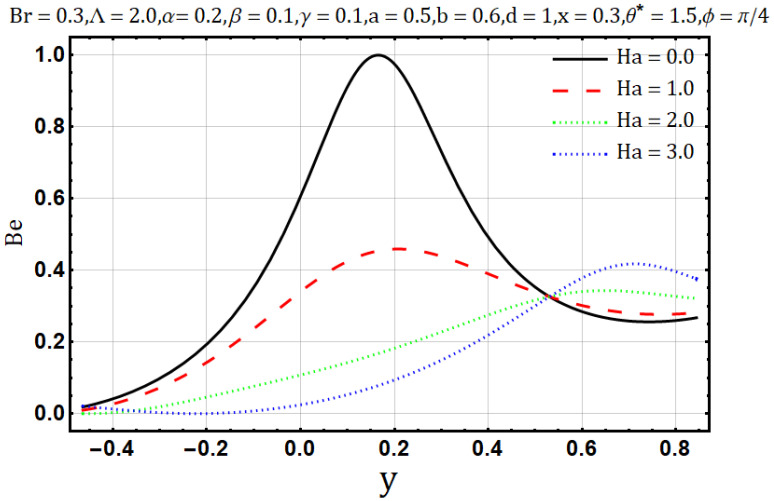
Velocity profile for viscosity parameter (α).

**Figure 7:**
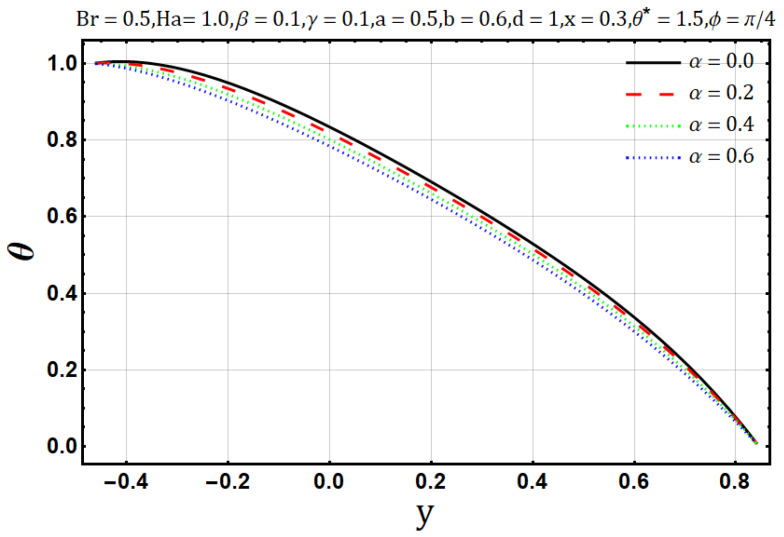
Temperature profile for viscosity parameter (α).

**Figure 8:**
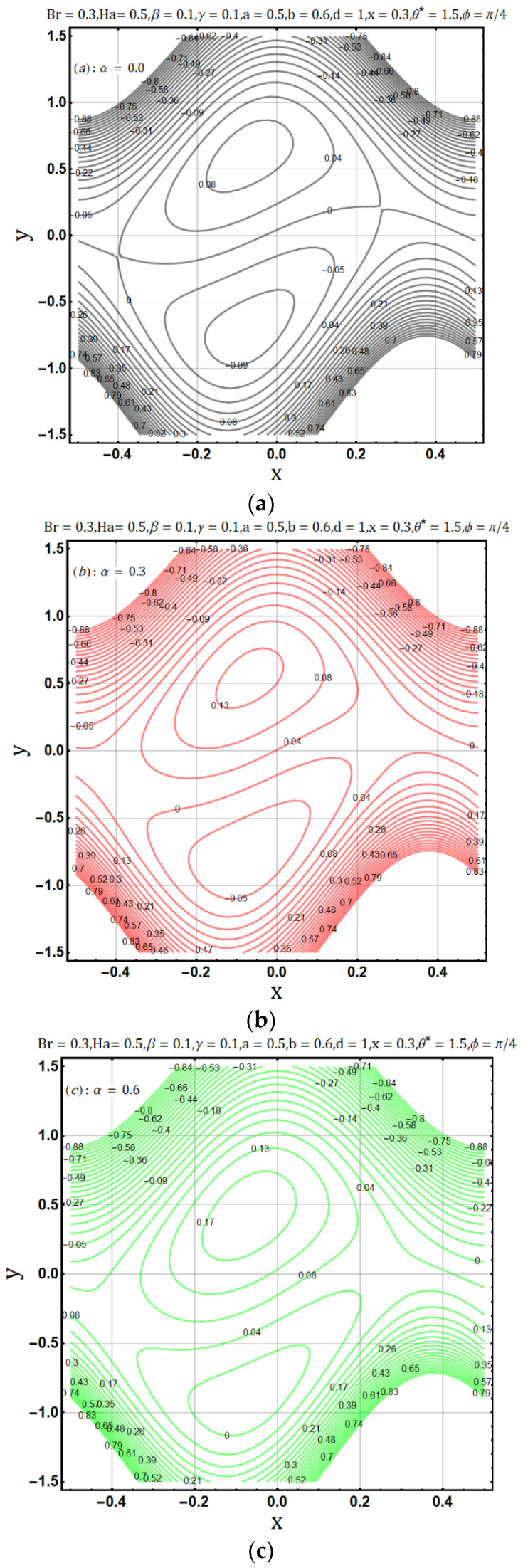
(**a**): Effect of viscosity parameter (α  =  0.0) on trapping. (**b**): Effect of viscosity parameter (α = 0.3) on trapping. (**c**): Effect of viscosity parameter (α = 0.6) on trapping.

**Figure 9:**
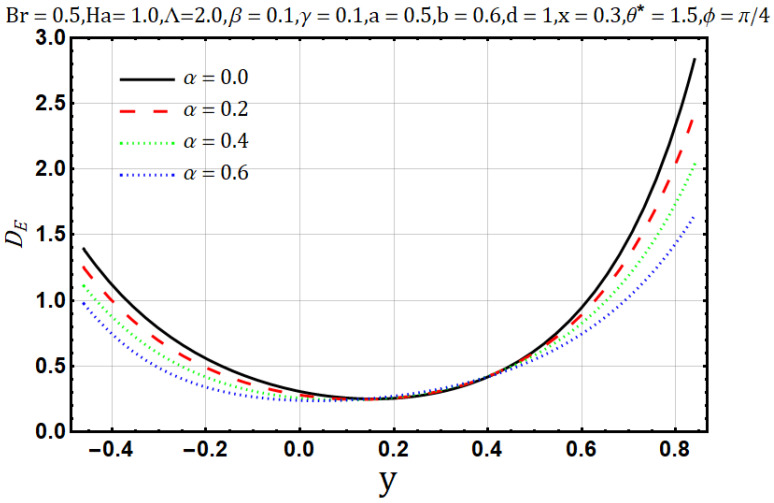
Entropy generation profile for viscosity parameter (α).

**Figure 10:**
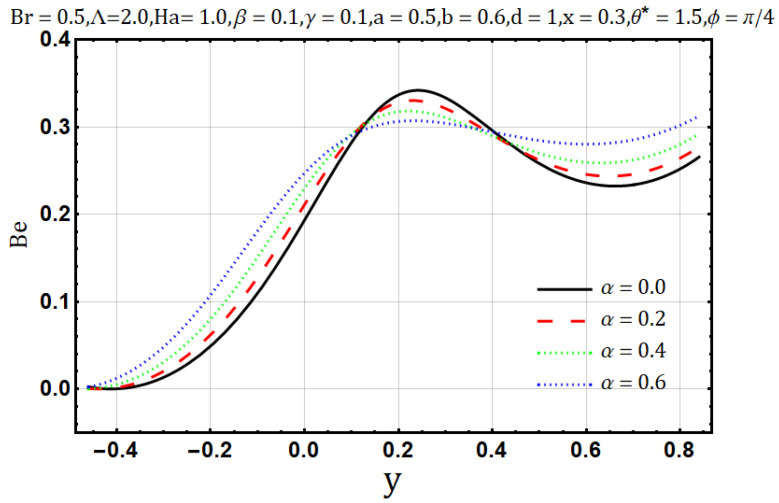
Bejan number profile for viscosity parameter (α).

Influence of variable viscosity parameter (α):

Viscosity offers resistance in the flow of heat and fluids, in Figure 6, as the variable viscosity parameter (α) increases the velocity initially increases but decreases later. In other words, velocity behaves oppositely near the upper and lower walls, but a negligible effect is observed in the center of the channel by the increase of variable viscosity parameter (α). Similarly, heat flow drops down when the viscosity measure increases, as shown in Figure 7. Streamline circulations get stronger as the variable viscosity parameter (α) increases, hence the size and number of boli increases subsequently, as shown in Figure 8a–c. In Figure 8 and Figure 9, entropy generation $\left(D_{E}\right)$ and Bejan number (Be) is seen with the increasing effect of variable viscosity parameter (α) respectively. It is noted that entropy generation $\left(D_{E}\right)$ is significantly controlled on the increase of variable viscosity parameter (α). Converse to the entropy generation $\left(D_{E}\right)$, Bejan number Be is seen to increase near the channel walls but is decreasing in the center as shown in Figure 10.

**Figure 11:**
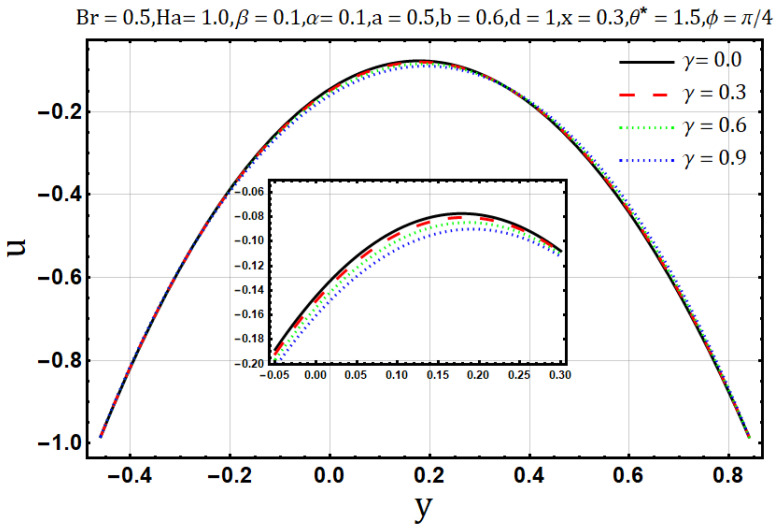
Velocity profile for electrical conductivity parameter (γ).

**Figure 12:**
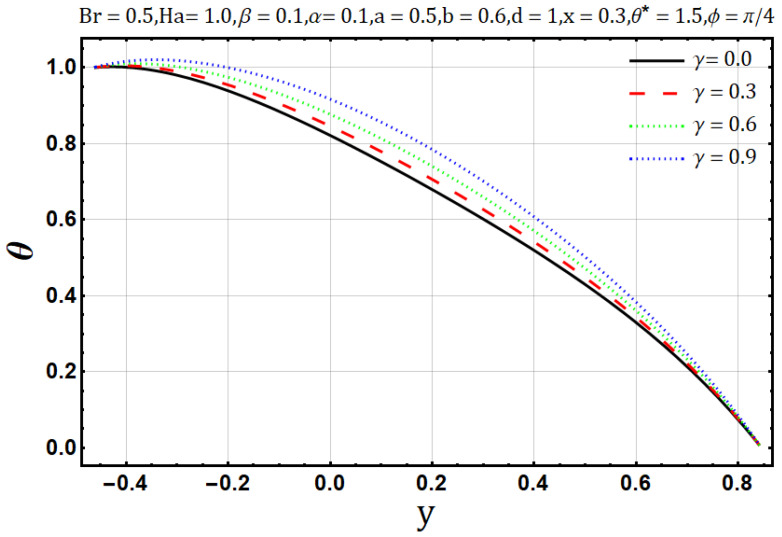
Temperature profile electrical conductivity parameter (γ).

**Figure 13:**
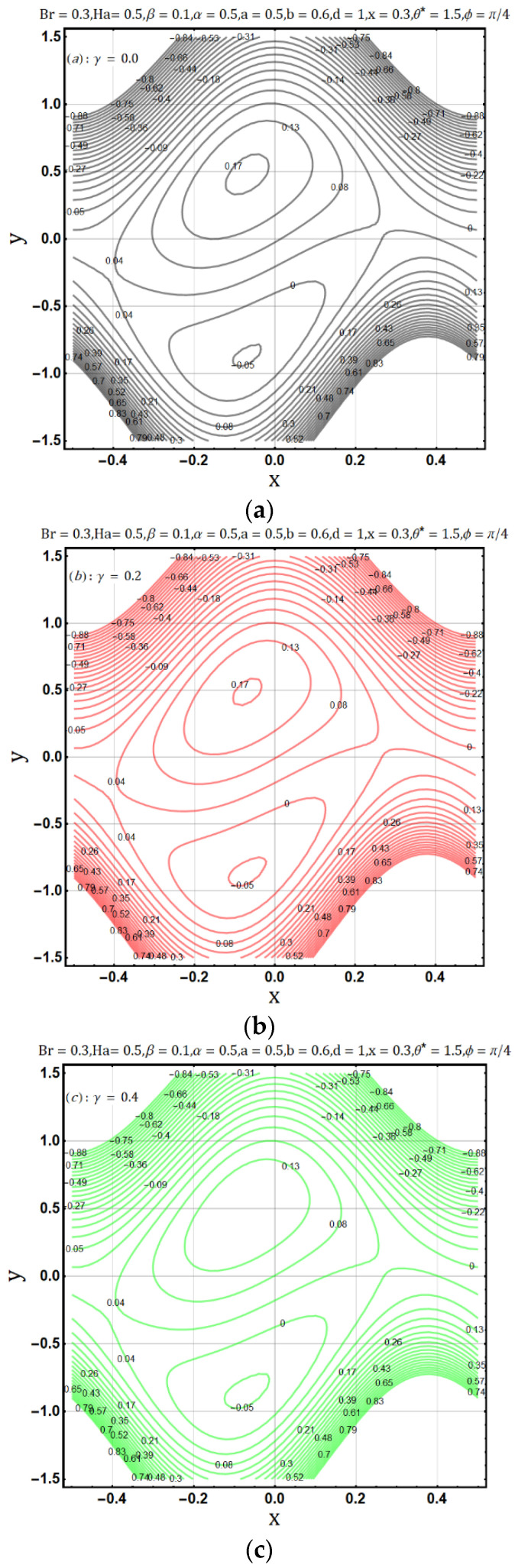
(**a**): Effect of electrical conductivity parameter (γ = 0.0) on trapping. (**b**): Effect of electrical conductivity parameter (γ = 0.2) on trapping. (**c**): Effect of electrical conductivity parameter (γ = 0.4) on trapping.

**Figure 14:**
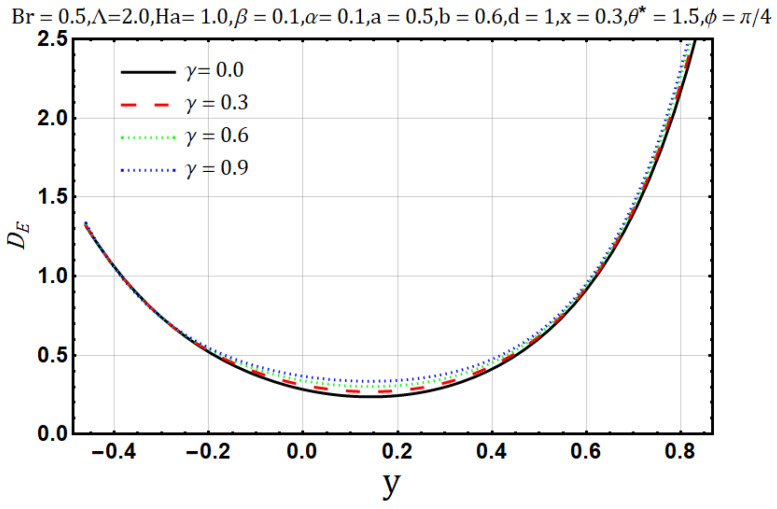
Entropy generation profile for electrical conductivity parameter (γ).

**Figure 15:**
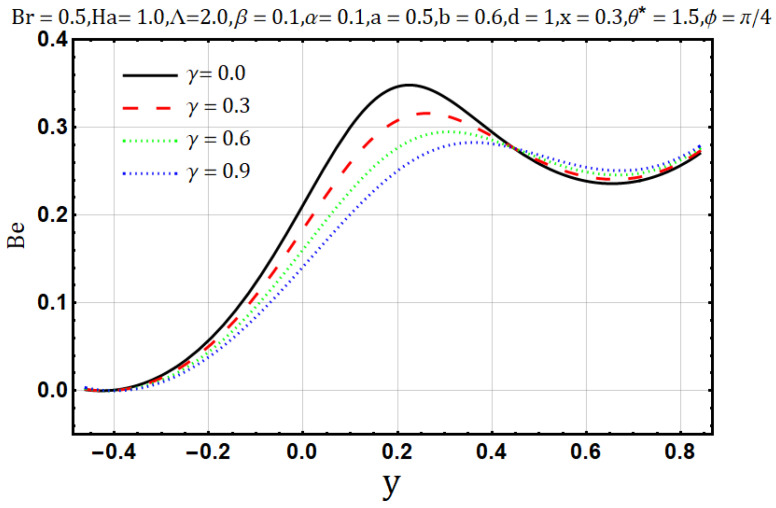
Bejan number profile for electrical conductivity parameter (γ).

Influence of variable electrical conductivity parameter (γ):

Electrical conductivity is the measure of degree to which a material conducts electricity. Increasing the electrical conductivity parameter (γ) may disturb the flow of heat and fluid as well; such effects are displayed in Figure 11 and Figure 12 Fluid flow reduces when variable electrical conductivity parameter (γ) is increased whereas the temperature profile shows the increasing behavior of heat flow on increasing the said parameter. In Figure 13 streamlines are viewed against the increments of variable electrical conductivity parameter (γ). The figure highlights that the trapping boli reduce both in size and number when the variable electrical conductivity parameter (γ) is increased. Variable electrical conductivity parameter (γ) considerably affects the behavior of entropy generation $\left(D_{E}\right)$ and Bejan number (Be). Both behave oppositely upon the increase of the said parameter; entropy generation increases while the Bejan number decreases (Figure 14 and Figure 15).

**Figure 16:**
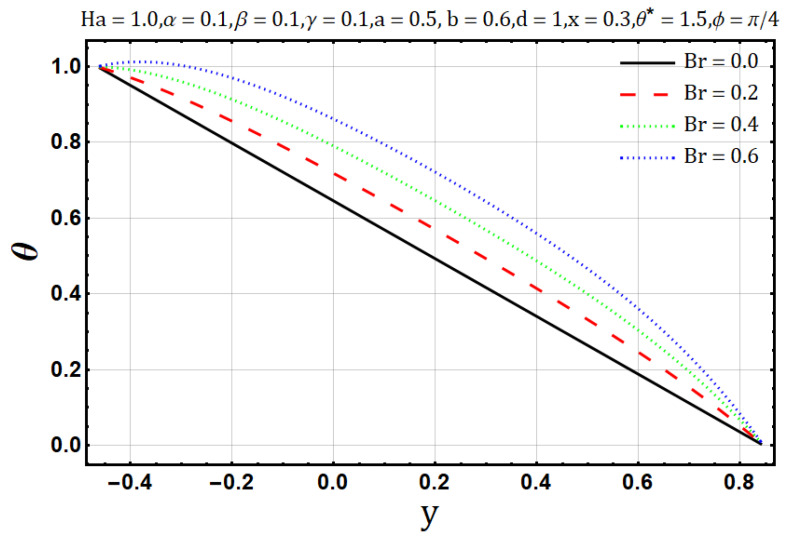
Temperature profile for Brinkman number (Br).

**Figure 17:**
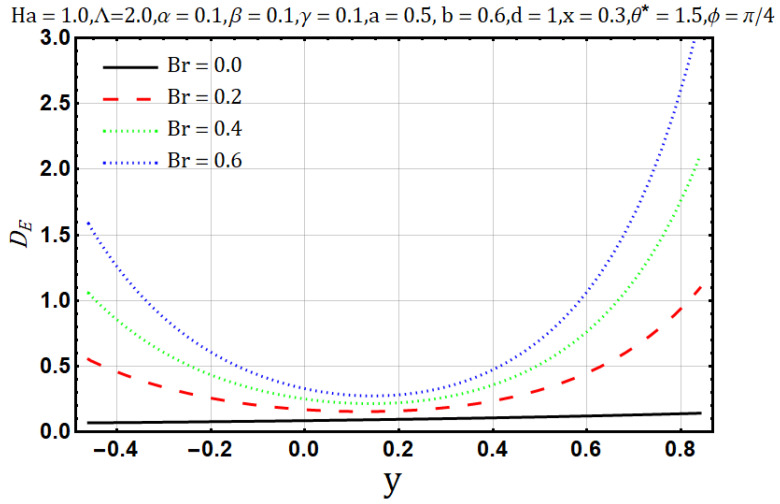
Entropy generation profile for Brinkman number (Br).

**Figure 18:**
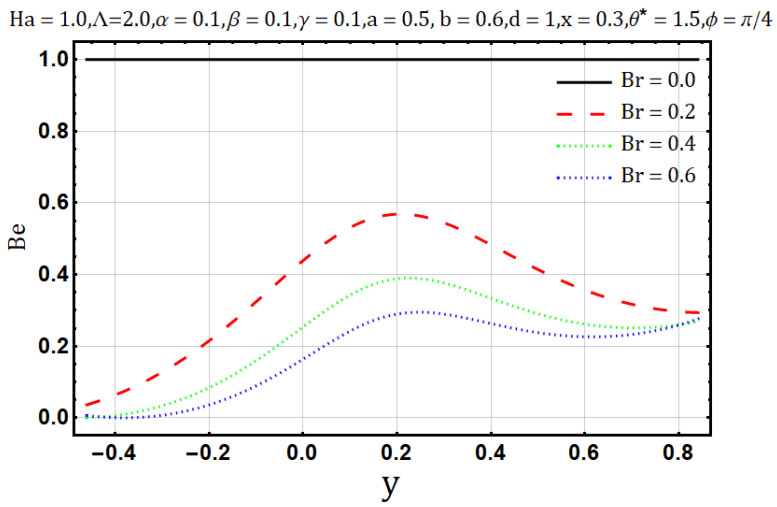
Bejan number profile for Brinkman number (Br).

Influence of Brinkman number (Br):

Brinkman number, in the fluid flow, characterizes the viscous dissipation as it is the ratio of viscous heat generation to the external heating. Figure 16 shows that the heat flow and Brinkman number are proportional to each other; the temperature profile tends to increase with the upsurge of Brinkman number (Br). Similar behavior is also reported for entropy generation as shown in Figure 17. On the contrary, Figure 18 shows that the Bejan number tends to decrease with the increase of Brinkman number (Br).

**Figure 19:**
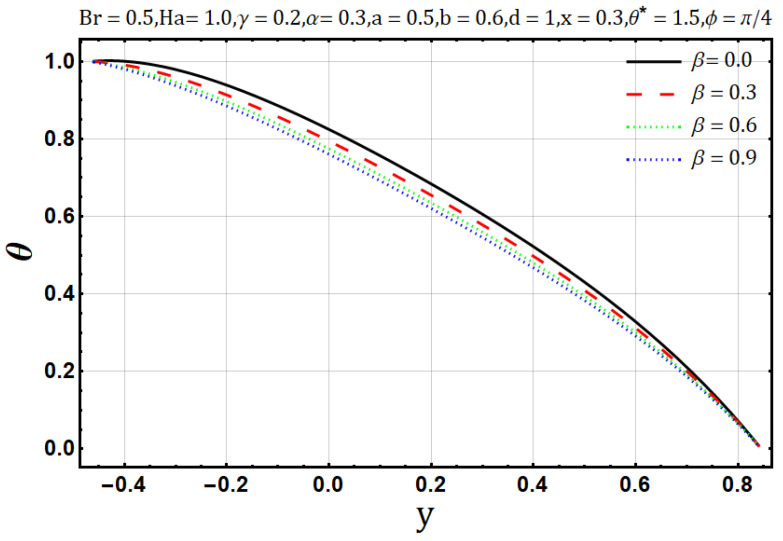
Temperature profile for thermal conductivity parameter (β).

**Figure 20:**
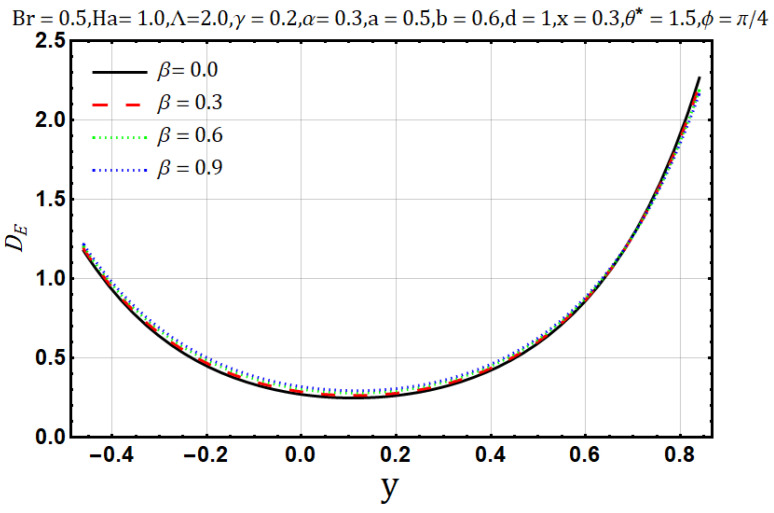
Entropy generation profile for thermal conductivity parameter (β).

**Figure 21:**
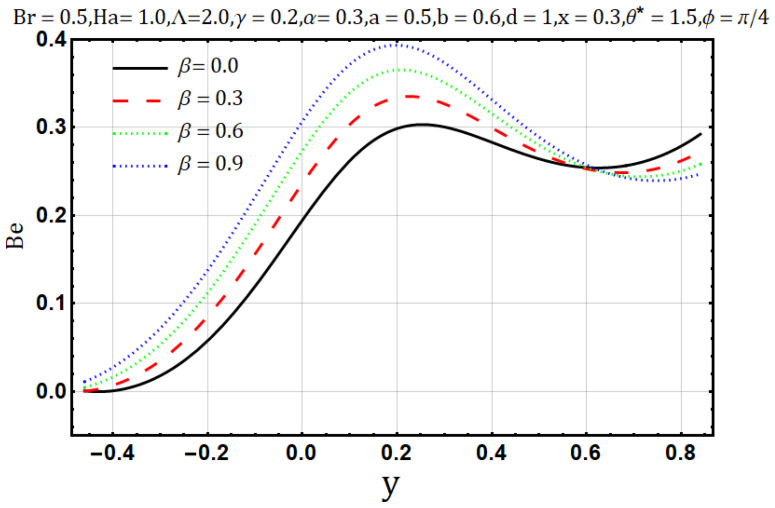
Bejan number profile for thermal conductivity parameter (β).

Influence of variable thermal conductivity parameter (β):

The heat conduction capability of materials is known as thermal conductivity (β). Naturally, heat flow reduces when the thermal conductivity increases. In Figure 19, the temperature profile decreases as the thermal conductivity parameter is increased. An increase in thermal conductivity (β) may cause disorder throughout the heat flow process; thus, it may cause entropy generation when thermal conductivity (β) of the fluids is increased. Figure 20 shows a direct relation between thermal conductivity (β) and entropy generation. Bejan number/irreversibility also behaves similarly, as does the entropy generation; in Figure 21, it increases upon the boost of thermal conductivity parameter (β).

## 5. Conclusions

All variable flow and heat properties are studied in an entropy analysis of MHD peristaltic flow of incompressible fluids within an asymmetric channel. Thermal analysis is also done with no slip conditions. Hence, the main results and outcomes can be summarized as follows:

- Fluid flow is observed to reduce when the Hartman number $\left(Ha^{2}\right)$, viscosity parameter (α) and electrical conductivity parameters (γ) are enhanced.
- Heat transfer is reported to behave similarly for variation of Hartman number $\left(Ha^{2}\right)$, electrical conductivity parameters (γ) and Brinkman number (Br) but it presents opposite behavior in the case of the viscosity parameter (α).
- Entropy generation is amplified by the variation of Hartman number $\left(Ha^{2}\right)$, electrical conductivity parameters (γ) and Brinkman number (Br).
- Bejan number is reduced when Hartman number $\left(Ha^{2}\right)$, electrical conductivity parameters (γ) and Brinkman number (Br) are enhanced.

## Figures and Tables

**Table 1:** Comparison between Present Results and the Results of Srinivas and Kothandapani [56] for the Ideal Heat Transfer Coefficient $Z\;=\;h_{1x}\;\theta_{y}\left(x,h_{1}\right)$, in the Special Case of Constant Fluid Properties (*α* = *β* = *γ* = 0) when Br = PrEc, b = 0.6, d = 1.5 and *ϕ* = π/4.

a	F	M	Br	x = 0.1		x = 0.2		x = 0.3	
				[56]	NDSolve	[56]	NDSolve	[56]	NDSolve
0.5	−2.0	2.0	1.0	1.0586	1.0586	1.4556	1.4556	1.7596	1.7596
0.7	−2.0	2.0	1.0	1.5418	1.5418	2.0650	2.0650	2.6909	2.6909
0.9	−2.0	2.0	1.0	2.0542	2.0542	2.6953	2.6953	3.8524	3.8524
1.1	−2.0	2.0	1.0	2.5920	2.5920	3.3484	3.3484	5.3457	5.3457
0.5	−0.5	2.0	3.0	8.9333	8.9333	15.9061	15.9061	17.4565	17.4565
0.5	−1.0	2.0	3.0	5.9373	5.9373	9.1554	9.1554	7.6982	7.6982
0.5	−1.5	2.0	3.0	3.6075	3.6075	4.4727	4.4727	2.5390	2.5390
0.5	−2.0	2.0	3.0	1.9440	1.9440	1.8582	1.8582	1.9789	1.9789
0.5	−2.0	0.0	3.0	1.8449	1.8449	1.8352	1.8352	1.9738	1.9738
0.5	−2.0	2.0	3.0	1.9440	1.9440	1.8582	1.8582	1.9789	1.9789
0.5	−2.0	3.0	3.0	2.1353	2.1353	1.9120	1.9120	1.9932	1.9932
0.5	−2.0	4.0	3.0	2.3848	2.3848	1.9920	1.9920	2.0182	2.0182
0.5	−2.0	2.0	1.0	1.0586	1.0586	1.4556	1.4556	1.7596	1.7596
0.5	−2.0	2.0	2.0	1.5013	1.5013	1.6569	1.6569	1.8692	1.8692
0.5	−2.0	2.0	3.0	1.9440	1.9440	1.8582	1.8582	1.9789	1.9789
0.5	−2.0	2.0	4.0	2.3868	2.3868	2.0595	2.0595	2.0885	2.0885

**Table 2:** Effects of Different Physical Parameters on Heat Transfer Rate $w_{1x}\theta_{y}\left(x,w_{1}\right),\;\;$ when a = 0.5, b = 0.6, d = 1.0, x = 0.3 and θ* = 1.5. Comparison between NDSolve and bvp4c.

Parameters					$h_{1x}\theta_{y}\left(x,h_{1}\right)$	
M	Br	α	β	γ	NDSolve	bvp4c
0.0	0.3	0.2	0.1	0.1	3.69812	3.69811
1.0	0.3	0.2	0.1	0.1	3.93678	3.93676
2.0	0.3	0.2	0.1	0.1	4.65001	4.65001
0.5	0.0	0.2	0.1	0.1	2.37393	2.37393
0.5	0.1	0.2	0.1	0.1	2.83928	2.83928
0.5	0.3	0.2	0.1	0.1	3.30056	3.30057
0.5	0.3	0.0	0.1	0.1	3.91107	3.91107
0.5	0.3	0.2	0.1	0.1	3.75783	3.75783
0.5	0.3	0.4	0.1	0.1	3.59546	3.59544
0.5	0.3	0.3	0.0	0.1	3.71009	3.71007
0.5	0.3	0.3	0.1	0.1	3.67806	3.67803
0.5	0.3	0.3	0.2	0.1	3.64911	3.64898
0.5	0.3	0.4	0.2	0.0	3.56551	3.56549
0.5	0.3	0.4	0.2	0.3	3.57507	3.57504
0.5	0.3	0.4	0.2	0.6	3.58466	3.58463
